# Succinic acid production from xylose mother liquor by recombinant *Escherichia coli* strain

**DOI:** 10.1080/13102818.2014.952501

**Published:** 2014-11-03

**Authors:** Honghui Wang, Jiachuan Pan, Jing Wang, Nan Wang, Jie Zhang, Qiang Li, Dan Wang, Xiaohua Zhou

**Affiliations:** ^a^ College of Chemistry and Chemical Engineering, Chongqing University, Chongqing400044, China; ^b^ Department of Biomedical and Chemical Engineering, Syracuse University, Syracuse, NY13244, USA; ^c^ National Key Laboratory of Biochemical Engineering, Institute of Process Engineering, Chinese Academy of Sciences, Beijing100190, China

**Keywords:** succinic acid, xylose mother liquor, response surface methodology, fermentation, *Escherichia coli*

## Abstract

Succinic acid (1,4-butanedioic acid) is identified as one of important building-block chemicals. Xylose mother liquor is an abundant industrial residue in xylitol biorefining industry. In this study, xylose mother liquor was utilized to produce succinic acid by recombinant *Escherichia coli* strain SD121, and the response surface methodology was used to optimize the fermentation media. The optimal conditions of succinic acid fermentation were as follows: 82.62 g L^−1^ total initial sugars, 42.27 g L^−1^ MgCO_3_ and 17.84 g L^−1^ yeast extract. The maximum production of succinic acid was 52.09 ± 0.21 g L^−1^ after 84 h with a yield of 0.63 ± 0.03 g g^−1^ total sugar, approaching the predicted value (53.18 g L^−1^). It was 1.78-fold of the production of that obtained with the basic medium. This was the first report on succinic acid production from xylose mother liquor by recombinant *E. coli* strains with media optimization using response surface methodology. This work suggested that the xylose mother liquor could be an alternative substrate for the economical production of succinic acid by recombinant *E. coli* strains.

## Introduction

Succinic acid, a C_4_-dicarboxylic acid also known as amber acid, is an intermediate in the tricarboxylic acid cycle (TCA) or as an end product of anaerobic metabolism.[1] Succinic acid is classified as the most promising one among 12 platform bio-based chemicals by the US Department of Energy.[2] It is used as a special chemical in food, agricultural and pharmaceutical industries and is a potential platform chemical for the production of various high value-added derivatives, including 1,4-butanediol (BDO), tetrahydrofuran (THF), g-butyrolactone (GBL), succinimide and especially the biodegradable material poly-butylenessuccinate (PBS).[3,4] Traditionally, succinic acid is produced via a petroleum-based process, which leads to many problems, such as resource depletion, environmental pollution and higher cost.[5] Biomass is one of the most abundant and low-cost renewable resource. The interest in producing succinic acid from natural biomass has increased during the last decades,[6] while microbial fermentation of biomass to succinic acid has been testified as an environmentally friendly and energy-efficient process.[1,7] Major strains used to produce succinic acid include *Actinobacillus succinogenes*,[8] *Mannheimia succiniciproducens*,[9] *Anaerobiospirillum succiniciproducen*,[10] and recombinant *Escherichia coli*,[11,12] *Corynebacterium glutamicum*, *Saccharomyces cerevisiae* and *Basfia succiniciproducens*. However, the critical factor for its competition with petroleum-based succinic acid is the fermentation cost of bio-based succinic acid. Therefore, many agricultural and industrial residues have been used for the production of succinic acid as raw carbon resources to decrease the fermentation cost, such as whey, cane molasses, straw and wood hydrolysate.[13–15] Kim et al. have used wood hydrolysate-based medium as the culture medium to produce succinic acid by *M. succniciproducens* MBEL55E and 11.73 g L^−1^ final titre was obtained with a yield of 0.56 g g^−1^.[16] Li et al. carried out anaerobic batch cultivation with cotton stalk hydrolysates by *A. ssuccinogenes* BE-1, and finally, 15.8 g L^−1^ succinic acid concentration with a high yield of 1.23 g g^−1^ glucose was attained.[17] Thakker et al. utilized soluble carbohydrates of the soybean biomass to produce succinate, and a final succinate production of 312 mmol L^−1^ with a molar yield of 0.82 mol mol^−1^ hexose was gained by *E. coli* strain HL27659k(pkk313)(pRU600).[18] Wang et al. also indicated that the aqueous phase bio-oil (AP-bio-oil), the by-product of biorefinery, could be utilized by *E. coli* for cell growth and succinic acid production.[19]

Xylose mother liquor is an abundant by-product of acid hydrolysis in the process of xylose manufacture from corncob or sugarcane bagasse. More than 50,000 metric tons of xylose mother liquor was produced in 2009 in China,[20] and only a few of them were used to prepare low value-added products such as caramel and glycerine substitute for the toothpaste industry.[21] Most of them were discharged directly, which caused serious environment pollution and decreased the economic benefits of the xylitol industry. In recent years, the biotransformation leads to more attractive approaches for producing high-value compounds from xylose mother liquor, such as 2,3-butanediol, acetone, butanol and ethanol.[22] Xylose mother liquor is a viscous reddish-brown substance, which contains 35%–40% xylose, 10%–15% L-arabinose, 8%–10% glucose and 8%–10% D-galactose.[20,21] Hence, xylose mother liquor could be used as a sugar feedstock to produce succinic acid and decrease the fermentation cost. The recombinant *E. coli* SD121 constructed in our lab grows well in the medium containing xylose, L-arabinose, glucose and other trace amount of sugars to produce succinic acid.[23] To the best of our knowledge, utilizing xylose mother liquor to produce succinic acid by engineered *E. coli* strains is not reported yet.

To achieve high-level production of valuable fermentative products, it is important to optimize the fermentation conditions. The statistical experimental method of response surface methodology (RSM) is a useful tool that can be used to evaluate and analyse the interactions between different factors compared with the traditional one-factor-at-a-time method in microbial process optimization.[24,25] Meanwhile, RSM can be used to design and test the experiment, recognize and optimize the significant factors, identify the optimum conditions, establish the numerical correlations for the target responses,[26] and finally optimize the fermentation process. There are literature reports about optimization of succinic acid production with individual sugar as a carbon source using RSM [27,28]; however, the same was not done for the mixed sugars available in the hydrolysate for succinic acid production.

In this study, an industrial residue – xylose mother liquor – was used to produce succinic acid by anaerobic fermentation. Recombinant *E. coli* strain SD121 and the RSM were used to optimize the fermentation conditions and to achieve high-level production of valuable fermentative products.

## Materials and methods

### Strain, growth conditions and fermentation mediums

The *E. coli* SD121 strain was used for succinic acid fermentation.[23] *Escherichia coli* SD121 was constructed by introduction of the plasmid pTrc-cppc into DC1515 to overexpress the *ppc* gene from *Anabaena sp*.7120. DC1515 [*pflB*::Cam *ldhA*::Tn10 *ptsG*400::Kan in W1485] was kindly provided by Prof Clark, Southern Illinois University. Luria–Bertani (LB) medium with 50 μg mL^−1^ ampicillin antibiotic was used for the aerobic growth. A single colony was inoculated in 5 mL LB broth with ampicillin and cultured overnight. Then the seed inoculums were added to a 100 mL flask containing 20 mL of LB medium with ampicillin and cultured at 37 °C and 170 rpm until the OD_600_ reached approximately 1.0. Then the strain was inoculated in a sealed serum bottle for anaerobic fermentation. The basic fermentation medium contained (per litre): 60 g total sugars, 20 g tryptone, 10 g yeast extract, 0.3 g MgSO_4_•7H_2_O, 0.45 g Na_2_HPO_4_•12H_2_O, 0.6 g NaH_2_PO•2H_2_O, 0.3 g (NH_4_)_2_SO_4_•7H_2_O, 0.2 g CaCl_2_ and 20 g MgCO_3_. 0.1 mmol L^−1^ IPTG was added after 4 h of an aerobic culture to induce *ppc* expression. The initial pH of the medium was adjusted to 7.0 with 10 mol L^−1^ NaOH solution and 10% H_2_SO_4_ (v/v). The medium was inoculated with 5% seed inoculums, cultured at 37 °C and 170 rpm for 84 h.

### Treatment of xylose mother liquor

The xylose mother liquor was given as a gift from Sichuan ZhongMu Feed Manufacturing Co. Ltd in China. It contains two major inhibitors: furfural and 5-hydroxymethylfurfural (HMF), which has deleterious effects on the growth of the strains [20] and decreases the production of succinic acid.

In this study, the method of overliming (Ca(OH)_2_ treatments) [29,30] was used to reduce the concentration of furfural and HMF as follows: 40 g xylose mother liquor was added to 200 mL H_2_O and stirred at room temperature for 1 h; afterwards, the pH was adjusted to 10.0 with anhydrous Ca(OH)_2_ followed by sedimentation for 1 h; then the supernatant was filtrated and decanted. The pH was adjusted to 5.5 with 5 mol L^−1^ H_2_SO_4_ and Na_2_SO_3_ was added to the supernatant at 0.1 g L^−1^. After 15 min of boiling, the mixture was cooled to ambient temperature, then filtrated and preserved at 4 °C.

### Experiment design and data analysis

In order to identify the key factor that influenced succinic acid production, the effective technique for medium optimization of Plackett–Burman Design (PBD) was chosen, which had a two-factor design. The levels of 12 variables viz. the concentrations of total sugars in xylose mother liquor (A), MgCO_3_ (B), MgSO_4_•7H_2_O (C), tryptone (D), yeast extract (E), Na_2_HPO_4_•12H_2_O (F), NaH_2_PO•2H_2_O (G), (NH_4_)_2_SO_4_•7H_2_O (H) and CaCl_2_ (I) were selected for PBD study as listed in Table 1. To estimate the population standard error, two dummy variables without level change were included. All above levels were examined to determine the key factors affecting the production of succinic acid. The results of PBD are listed in Tables 1 and  2.

**Table 1.** Factor levels of PBD for screening of significant variables affecting succinic acid production and corresponding results.^a^

Run no.	A	B	C	D	E	F	G	H	I	Succinic acid production (g L^−1^)
1	−1 (50)^b^	−1 (20)	−1 (0.3)	−1 (20)	−1 (8)	−1 (0.3)	−1 (4)	−1 (2)	−1 (0.2)	26.65 ± 0.28 ^c^
2	1 (70)	−1 (20)	1 (0.5)	1 (25)	1 (12)	−1 (0.3)	−1 (4)	−1 (2)	1 (0.4)	32.52 ± 0.42
3	1 (70)	1 (30)	−1 (0.3)	−1 (20)	−1 (8)	1 (0.6)	−1 (4)	1 (4)	1 (0.4)	38.61 ± 0.31
4	−1 (50)	−1 (20)	1 (0.5)	−1 (20)	1 (12)	1 (0.6)	−1 (4)	1 (4)	1 (0.4)	31.11 ± 0.41
5	−1 (50)	1 (30)	1 (0.5)	1 (25)	−1 (8)	−1 (0.3)	−1 (4)	1 (4)	−1 (0.2)	29.57 ± 0.25
6	1 (70)	−1 (20)	−1 (0.3)	−1 (20)	1 (12)	−1 (0.3)	1 (7)	1 (4)	−1 (0.2)	32.82 ± 0.11
7	1 (70)	−1 (20)	1 (0.5)	1 (25)	−1 (8)	1 (0.6)	1 (7)	1 (4)	−1 (0.2)	28.49 ± 0.26
8	1 (70)	1 (30)	1 (0.5)	−1 (20)	−1 (8)	−1 (0.3)	1 (7)	−1 (2)	1 (0.4)	31.67 ± 0.28
9	−1 (50)	−1 (20)	−1 (0.3)	1 (25)	−1 (8)	1 (0.6)	1 (7)	−1 (2)	1 (0.4)	27.81 ± 0.33
10	1 (50)	1 (30)	−1 (0.3)	1 (25)	1 (12)	1 (0.6)	−1 (4)	−1 (2)	−1 (0.2)	41.64 ± 0.48
11	−1 (50)	1 (30)	−1 (0.3)	1 (25)	1 (12)	−1 (0.3)	1 (7)	1 (4)	1 (0.4)	31.52 ± 0.39
12	−1 (50)	1 (30)	1 (0.5)	−1 (20)	1 (12)	1 (0.6)	1 (7)	−1 (2)	−1 (0.2)	32.33 ± 0.35

^a^The two dummy variables are not shown.

^b^Real values (g L^−1^) of the factors and the corresponding coded level.

^c^Experiments were performed in triplicates.

**Table 2.** ANOVA for selected factorial model of PBD.

Source	Sum of squares	Degrees of freedom	Mean square	*F*-value	*p*-value Prob > *F*
Model	200.92	9	22.32	19.91	0.0487
A	59.67	1	59.67	53.23	0.0183
B	56.07	1	56.07	50.02	0.0194
C	14.87	1	14.87	13.27	0.0678
D	0.22	1	0.22	0.2	0.6985
E	30.53	1	30.53	27.23	0.0348
F	19.35	1	19.35	17.26	0.0533
G	19.92	1	19.92	17.77	0.0519
H	0.021	1	0.021	0.019	0.9041
I	0.25	1	0.25	0.23	0.682
Residual	2.24	2	1.12		
Cor total	203.16	11			

Based on the results of PBD, the key factors that significantly influenced succinic acid production were identified, and then the steepest ascent (SA) design was used to move rapidly towards the optimum response.[31] The experimental design and results of SA are shown in Table 3.

**Table 3.** Experimental design and results of SA.

Run no.	Concentration of total sugars (g L^−1^)	MgCO_3_ (g L^−1^)	Yeast extract (g L^−1^)	Yield (g L^−1^)
1	70	30	12	40.27 ± 0.34 ^a^
2	75	35	14	43.54 ± 0.27
3	80	40	16	49.31 ± 0.31
4	85	45	18	42.76 ± 0.42
5	90	50	20	34.92 ± 0.18

^a^Experiments were performed in triplicates.

To optimize the screened variables for improving the production of succinic acid, a Box–Behnken Design (BBD) with three coded levels was performed, and the experiment design and results are shown in Table 4. The experimental data were analysed by RSM using the mathematical model developed as the following second-order polynomial equation in RSM of BBD to predict the optimal point:(1) 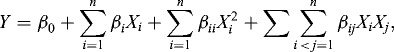

**Table 4.** Design matrix of BBD and corresponding results.

Run no.	X_1_	X_2_	X_3_	Actual value (g L^−1^)	Predicted value (g L^−1^)
1	−1 (75) ^a^	−1 (35)	0 (16)	39.33 ± 0.34 ^b^	39.54
2	1 (85)	−1 (35)	0 (16)	42.18 ± 0.27	42.36
3	−1 (75)	1 (45)	0 (16)	44.53 ± 0.31	44.35
4	1 (85)	1 (45)	0 (16)	47.01 ± 0.26	46.80
5	−1 (75)	0 (40)	−1 (14)	39.54 ± 0.45	39.51
6	1 (85)	0 (40)	−1 (14)	40.23 ± 0.23	40.24
7	−1 (75)	0 (40)	1 (18)	47.15 ± 0.34	47.15
8	1 (85)	0 (40)	1 (18)	51.64 ± 0.44	51.68
9	0 (80)	−1 (35)	−1 (14)	37.28 ± 0.21	37.10
10	0 (80)	1 (45)	−1 (14)	36.91 ± 0.37	37.12
11	0 (80)	−1 (35)	1 (18)	42.24 ± 0.26	42.03
12	0 (80)	1 (45)	1 (18)	51.08 ± 0.32	51.26
13	0 (80)	0 (40)	0 (16)	50.13 ± 0.42	50.12
14	0 (80)	0 (40)	0 (16)	49.98 ± 0.23	50.12
15	0 (80)	0 (40)	0 (16)	50.25 ± 0.19	50.12

^a^Real values (g L^−1^) of independent variables and the corresponding coded level.

^b^Experiments were performed in triplicates.

where *Y* is the predicted response (succinic acid production, g L^−1^), 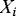 and 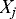 are the coded independent variables, 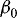 is the intercept, and 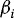, 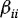 and 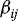 are the constant coefficients of the linear effect, quadratic effect and interaction effect, respectively.

Design-Expert 8.0 Stat-Ease, Inc. (Minneapolis, USA) was used for regression analysis, analysis of variance (ANOVA) and graphical optimization.

### Validation of the model

Validation was carried out under the optimized conditions from the above calculations to determine the accuracy of the model, and the conditions were further verified by experiments performed in triplicate.

### Analytical methods

The fermentation production and different reductive sugar were analysed by HPLC using an Aminex HPX-87H ion-exchange column (Bio**-**Rad, USA) and HP1200 chromatography station system (Agilent Technologies, USA) equipped with an ultraviolet absorbance detector (Agilent Technologies, G1315D) and a refractive index detector (Agilent Technologies, G1362A). A mobile phase of 5 mmol L^−1^ H_2_SO_4_ solution at a 0.6 mL min^−1^ flow rate was used at 55 °C. The injection volume was 20 μl, and the detection wave was set at 210 nm.

## Results and discussion

### Fermentation of xylose, L-arabinose, glucose and galactose

In this study, we examined *E. coli* SD121 to see how it is able to produce succinic acid using various concentrations of xylose, L-arabinose, glucose and galactose individually before utilizing a mixture of these sugars (found in the xylose mother liquor).

**Figure 1.**
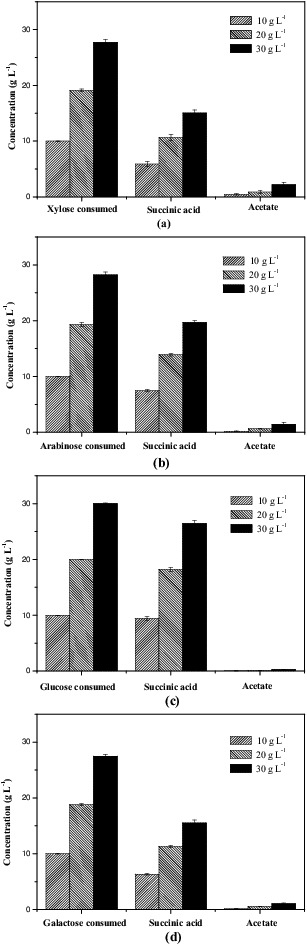
Sugar metabolism by recombinant *E. coli* SD121 at 48 h. (a) Xylose consumed and metabolites produced; (b) L-arabinose consumed and metabolites produced; (c) glucose consumed and metabolites produced; (d) galactose consumed and metabolites produced. The initial concentrations of sugars were in the range of 10–30 g L^−1^.

The sugars metabolism and succinic acid production by *E. coli* SD121 were examined using different concentrations of initial sugars in the range of 10–30 g L^−1^. The strain SD121 consumed 10.00, 19.10 and 27.70 g L^−1^ xylose in 48 h and produced 5.89, 10.67 and 15.06 g L^−1^ succinic acid with yields of 0.59, 0.56 and 0.54 g g^−1^ xylose while using 10, 20 and 30 g L^−1^ initial xylose, respectively (Figure 1 (a)). The strain SD121 consumed 10.00, 19.30 and 28.20 g L^−1^ arabinose in 48 h and produced 7.44, 13.90 and 19.68 g L^−1^ succinic acid with yields of 0.74, 0.72 and 0.69 g g^−1^ arabinose while using 10, 20 and 30 g L^−1^ initial arabinose, respectively (Figure 1 (b)). The strain SD121 consumed 10.00, 20.00 and 30.00 g L^−1^ glucose in 48 h and produced 9.45, 18.21 and 26.44 g L^−1^ succinic acid with yields of 0.95, 0.91 and 0.88 g g^−1^ glucose while using 10, 20 and 30 g L^−1^ initial glucose (Figure 1 (c)). Moreover, the strain SD121 consumed 10.00, 18.80 and 27.50 g L^−1^ galactose in 48 h and produced 6.34, 11.29 and 15.52 g L^−1^ succinic acid with yields of 0.63, 0.60 and 0.56 g g^−1^ galactose while using 10, 20 and 30 g L^−1^ galactose, respectively (Figure 1 (d)). Trace amount of by-product acetate was observed for both sugars, and with increasing initial sugars concentration, succinic acid production increased, succinic acid yield decreased in both experiments. Results of the above experiments and previous researches [23] showed the ability of *E. coli* SD121 to efficiently metabolize xylose, L-arabinose, glucose and galactose to produce succinic acid.

### Optimization of succinic acid production using xylose mother liquor

The xylose mother liquor was treated with the method of overliming,[29,30] and then it was used as the carbon source for the production of succinic acid. The reducing sugar concentration detected by HPLC contained (per litre): 394.4 g xylose, 127.4 g L-arabinose, 104.7 g glucose, 85.3 g galactose and a trace of unknown sugars (probably mannose, rhamnose and oligosaccharides). The strain SD121 had already shown an ability to ferment such sugars and produce succinic acid, and in this study was explored for xylose mother liquor fermentation.

The PBD was applied to differentiate significant factors from a multivariable system. Table 1 presents the design matrix and experimental results for succinic acid production from the 12 trials PBD with two levels to evaluate the key factors of nine medium components. The results were subjected to regression analysis and ANOVA as shown in Table 2. The model *F*-value was 19.91 and there was only a 4.87% (Prob> *F* = 0.0487) chance that such a large ‘model *F*-Value’ could occur due to noise. Values of ‘Prob>*F*’ less than 0.0500, which indicated model terms are significant. In this case, *A*, *B* and *E* were significant model terms, and the significance of the nine medium components could be ranked as total sugars > MgCO_3_ > yeast extract > NaH_2_PO•2H_2_O > Na_2_HPO_4_•12H_2_O > MgSO_4_•7H_2_O > CaCl_2_ > tryptone > (NH_4_)_2_SO_4_•7H_2_O. From the data analysis, sugars, MgCO_3_, yeast extract, Na_2_HPO_4_•12H_2_O and CaCl_2_ displayed a positive effect on succinic acid production, whereas NaH_2_PO•2H_2_O, MgSO_4_•7H_2_O and tryptone were found with a negative effect. Thus, the medium obtained after PBD had the following composition (per litre): 20 g tryptone, 0.3 g MgSO_4_•7H_2_O, 0.6 g Na_2_HPO_4_•12H_2_O, 4 g NaH_2_PO•2H_2_O, 2 g (NH_4_)_2_SO_4_•7H_2_O, 0.4 g CaCl_2_, and the other three factors were identified as significant variables and were applied in the optimization experiments that followed.

Based on the results of PBD, total sugars, MgCO_3_ and yeast extract were the key factors, and the high level was benefit for the succinic acid production from xylose mother liquor by recombinant *E. coli* SD121. Thus, the path of SA was moved along the path in which sugars, MgCO_3_ and yeast extract increased. The experimental design and results of SA are shown in Table 3. The highest succinic acid production of 49.31 g L^−1^ was observed under the point of levels: 80 g L^−1^ of total sugars, 40 g L^−1^ of MgCO_3_ and 16 g L^−1^ yeast extract, which indicated that this point was near the optimum and was chosen for the optimization experiments that followed.

The optimal level of the key factors: total sugars (*X*_1_), MgCO_3_ (*X*_2_) and yeast extract (*X*_3_) were selected for design of BBD. Based on the above results, the point (80 g L^−1^ of sugars, 40 g L^−1^ of MgCO_3_ and 16 g L^−1^yeast extract) was taken as the central level of BBD. Then the three-factor, three-level BBD was arranged as shown in Table 4. The design matrix of BBD and corresponding results (production of succinic acid, i.e. the response) along with the predicted response is summarized in Table 4.

The experimental results obtained from RSM were fitted to a second-order polynomial equation as follows (in terms of coded factors):(2) 
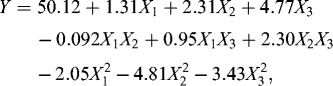
where *Y* is the predicted response (succinic acid production), and *X*_1_, *X*_2_, *X*_3_ are the coded values of sugars, MgCO_3_ and yeast extract, respectively.

Table 5 shows the ANOVA for the BBD. Values of ‘Prob > *F*’ less than 0.0500 indicated that model terms were significant and the values greater than 0.1000 indicated that the model terms were not significant. In this case *X*_1_, *X*_2_, *X*_3_, *X*_1_ *×*_2_, *X*_2_ *×*_3_, *X*_1_^2^, *X*_2_^2^ and *X*_3_^2^ were significant model terms. The ‘Lack of Fit *F*-value’ of 5.62 implied that the lack of fit was not significantly relative to the pure error. There is a 15.48% (Prob> *F* = 0.1548) chance that such a large ‘Lack of Fit *F*-value’ could occur due to noise. High model *F*-value (630.32) and non-significant lack of fit indicated that the model was a good fit. The ‘Pred *R* ^2^’ of 0.9872 was in reasonable agreement with the ‘Adj *R* ^2^’ of 0.9975. ‘Adeq Precision’ indicated the signal-to-noise ratio. A ratio greater than 4 was desirable,[32] and in this work the ratio of 67.938 indicated an adequate signal. This model could be used to navigate the design space.

**Table 5.** ANOVA for the Response Surface Quadratic Model.

Source	Sum of squares	Degrees of freedom	Mean square	*F*-value	*p*-value Prob > *F*
Model	391.57	9	43.51	630.32	<0.0001
*X*_1_	13.81	1	13.81	200.04	<0.0001
*X*_2_	42.78	1	42.78	619.80	<0.0001
*X*_3_	181.93	1	181.93	2635.68	<0.0001
*X*_1_×_2_	0.034	1	0.034	0.50	0.5128
*X*_1_×_3_	3.61	1	3.61	52.30	0.0008
*X*_2_×_3_	21.21	1	21.21	307.22	<0.0001
*X*_1_^2^	15.48	1	15.48	224.25	<0.0001
*X*_2_^2^	85.43	1	85.43	1237.60	<0.0001
*X*_3_^2^	43.50	1	43.50	630.25	<0.0001
Residual	0.35	5	0.069		
Lack of Fit	0.31	3	0.10	5.62	0.1548
Pure error	0.037	2	0.018		
Cor total	391.92	14			
*R*^2^ = 0.9991		Adj *R*^2^ = 0.9975		Pred *R*^2^ = 0.9872	
Adeq Precision = 67.938					

**Figure 2.**
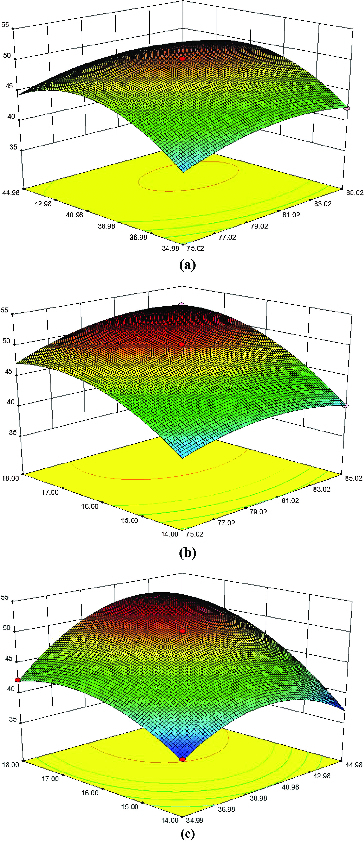
Response surface curves of succinic acid production by recombinant *E. coli* SD121 showing interaction between (a) total sugars and MgCO_3_, (b) total sugars and yeast extract and (c) MgCO_3_ and yeast extract.

To study the interactions between two variables and find out the optimum concentration of each factor for maximum succinic acid production, the three-dimensional response surface curves were depicted by keeping the other variables at zero level (Figure 2). Subsequently, the maximum succinic acid production was estimated from Equation (2) at the concentration of total sugars, MgCO_3_ and yeast extract of 82.62 g L^−1^, 42.27 g L^−1^ and 17.84 g L^−1^, respectively.

### Production of succinic acid using xylose mother liquor and validation of the model

**Figure 3.**
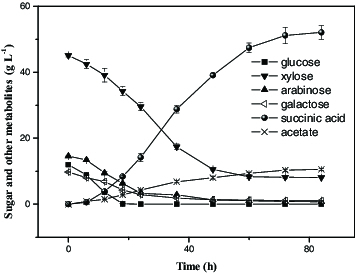
Fermentations using xylose mother liquor by recombinant *E. coli* SD121. Cells were grown in anaerobic bottles for 84 h with an initial total reductive sugar concentration of 82.62 g L^−1^. The error bars in the figure indicate the standard deviations (SD) among three parallel replicates.

To evaluate the validity of the quadratic model, the optimal medium compositions, namely, 82.62 g L^−1^ initial total sugars, 42.27 g L^−1^ MgCO_3_ and 17.84 g L^−1^ yeast extract, were used in an additional experiment in three replicates. Consequently, as shown in Figure 3, the strain SD121 consumed 37.01 g L^−1^ xylose, 13.86 g L^−1^ arabinose, 12.00 g L^−1^ glucose, 8.70 g L^−1^ galactose and a trace of unknown sugars in 84 h and the maximum production of succinic acid based on the xylose mother liquor was 52.09 ± 0.21 g L^−1^ after 84 hours with a yield of 0.63 ± 0.03 g g^−1^ total sugar, which agreed with the predicted value (53.18 g L^−1^) well, confirming the model's authenticity. The production was 1.78-fold of the production of that obtained with the basic medium, which was 29.83 g L^−1^. The production and final yield of succinic acid based on the xylose mother liquor were better than in previous reports (11.73 g L^−1^ and 0.56 g g^−1^) by Kim et al. who used wood hydrolysate-based medium as the carbon source,[16] and close to the data (57.81 g L^−1^ and 0.87 g g^−1^) reported by Wang et al. using corn stalk hydrolysates as the carbon source.[23] Acetate was the major by-product formed in the fermentation process, the final titre was about 10 g L^−1^ in 84 h. This study successfully suggested that the xylose mother liquor could be an alternative substrate for the economical production of succinic acid by recombinant *E. coli* strains. To further reduce the process cost, attempts can be made to replace complex and nitrogen source.

## Conclusions

Succinic acid production using an industrial residue – xylose mother liquor – by recombinant *E. coli* strain SD121 was demonstrated. The RSM was used to optimize the fermentation conditions, and a final succinic acid production of 52.09 ± 0.21 g L^−1^ after 84 hours was achieved. It showed a great potential use of xylose mother liquor as a feedstock for economical succinic acid production using recombinant *E. coli* strains. This is the first report to apply the RSM method to optimize the hydrolysate fermentation to produce succinic acid, which could be used in other hydrolysates fermentations to achieve an economical succinic acid production.
